# Tetra­ethyl­ammonium 12-phenyl­ethynylcarba-*closo*-dodeca­borate, [Et_4_N][12-PhCC-*closo*-CB_11_H_11_]

**DOI:** 10.1107/S1600536809013300

**Published:** 2009-04-18

**Authors:** Maik Finze, Guido J. Reiss

**Affiliations:** a Institut für Anorganische Chemie und Strukturchemie II, Heinrich-Heine-Universität Düsseldorf, Universitätsstrasse 1, D-40225 Düsseldorf, Germany

## Abstract

The asymmetric unit of the title compound, C_8_H_20_N^+^·C_9_H_16_B_11_^−^ or [Et_4_N][12-PhCC-*closo*-CB_11_H_11_], consists of one cation and one anion. The [12-PhCC-*closo*-CB_11_H_11_]^−^ anion is close to possessing a non-crystallographic plane of mirror symmetry with a nearly linear B—C C—C group, with B—C C and C C—C angles of 177.15 (16) and 176.64 (17)°, respectively.

## Related literature

Carba-*closo*-dodeca­borate anions with functional groups are potential building blocks for a variety of applications, for example ionic liquids and liquid crystals, see: Körbe *et al.* (2006 ▶). Recently, we have shown that {*closo*-CB_11_} clusters with one or two alkynyl groups bonded to boron are accessible by Pd-catalysed Kumada-type cross-coupling reactions starting from the corresponding mono- and diiodinated clusters, see: Finze (2008 ▶, 2009 ▶).
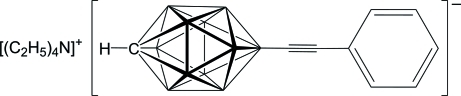


## Experimental

### 

#### Crystal data

C_8_H_20_N^+^·C_9_H_16_B_11_^−^
*M* *_r_* = 373.38
Triclinic, 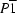

*a* = 8.8201 (6) Å
*b* = 12.0929 (11) Å
*c* = 12.1858 (11) Å
α = 81.032 (7)°
β = 79.899 (7)°
γ = 71.553 (7)°
*V* = 1206.82 (18) Å^3^
*Z* = 2
Mo *K*α radiation
μ = 0.05 mm^−1^
*T* = 293 K
0.30 × 0.25 × 0.20 mm

#### Data collection

Stoe Stadi CCD diffractometer
Absorption correction: none
17081 measured reflections
4219 independent reflections
3228 reflections with *I* > 2σ(*I*)
*R*_int_ = 0.051

#### Refinement

*R*[*F* ^2^ > 2σ(*F* ^2^)] = 0.048
*wR*(*F* ^2^) = 0.101
*S* = 1.00
4219 reflections
363 parameters
H atoms treated by a mixture of independent and constrained refinement
Δρ_max_ = 0.16 e Å^−3^
Δρ_min_ = −0.16 e Å^−3^

### 

Data collection: *CrysAlis CCD* (Kuma, 2000 ▶); cell refinement: *CrysAlis RED* (Kuma, 2000 ▶); data reduction: *CrysAlis RED*; program(s) used to solve structure: *SHELXS97* (Sheldrick, 2008 ▶); program(s) used to refine structure: *SHELXL97* (Sheldrick, 2008 ▶); molecular graphics: *DIAMOND* (Brandenburg, 2008 ▶); software used to prepare material for publication: *SHELXL97*.

## Supplementary Material

Crystal structure: contains datablocks I, global. DOI: 10.1107/S1600536809013300/br2103sup1.cif

Structure factors: contains datablocks I. DOI: 10.1107/S1600536809013300/br2103Isup2.hkl

Additional supplementary materials:  crystallographic information; 3D view; checkCIF report

## Figures and Tables

**Fig. 1.**
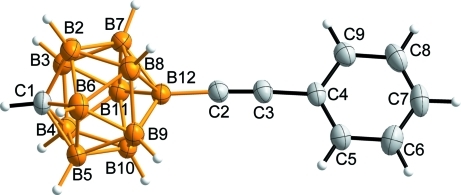
: The 12-phenylethinylcarba-closo-dodecaborate cation in [Et4N][12-PhCC-closo-CB11H11]. Hydrogen atoms are drawn with an arbitrary radius and the displacement ellipsoids are shown at the 40% probability level.

**Fig. 2.**
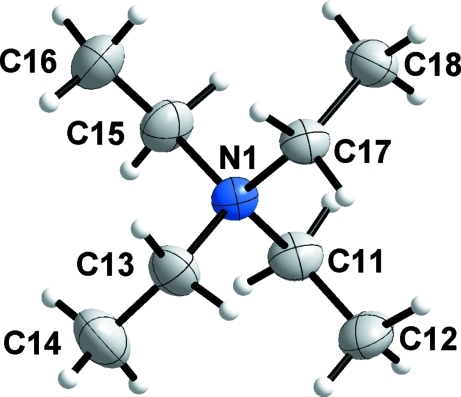
: The tetraethylammonium cation in [Et4N][12-PhCC-closo-CB11H11]. Hydrogen atoms are drawn with an arbitrary radius and the displacement ellipsoids are shown at the 40% probability level.

## supplementary crystallographic information

### Comment

Carba-*closo*-dodecaborate anions with functional groups are potential building blocks for a variety of applications, for example ionic liquids and liquid crystals (Körbe, 2006). Recently, we have shown that {*closo*-CB_11_} clusters with one or two alkynyl groups bonded to boron are accessible by Pd-catalyzed Kumada-type cross-coupling reactions starting from the corresponding mono- and diiodinated clusters, respectively (Finze, 2008, 2009). The title compound [Et_4_N][12-PhCC-*closo*-CB_11_H_11_] crystallizes in the triclinic centrosymmetric space group P1 with one formula unit in the asymmetric unit. The bond lengths and angles in the [12-PhCC-*closo*-CB_11_H_11_]^-^ anion in its [Et_4_N]^+^ salt are close to the values reported for the respective Cs^+^ salt (Finze, 2008). However, the quality of the data for the [Et_4_N]^+^ salt described herein is significantly better than the quality of the data obtained for the Cs^+^ salt. The thermal properties of [Et_4_N][12-PhCC-*closo*-CB_11_H_11_] were studied by differential scanning calorimetry (DSC). The salt melts at 433 K and is thermally stable up to 518 K.

### Experimental

[Et_4_N][12-PhCC-*closo*-CB_11_H_11_] was synthesized according to a published procedure (Finze, 2008). The spectroscopic data have been reported earlier (Finze, 2008). The salt was dissolved in acetonitrile and slow evaporation of the solvent resulted in colorless crystals.

### Refinement

All hydrogen atom positions were obtained from difference fourier maps. The hydrogen atoms of the methyl groups were included in the latest stages of the refinement with a riding model and for each methyl group a common *U*_iso_ value was refined. The positional parameters and the isotropic displacement parameters of all other hydrogen atoms were refined freely.

### Crystal data

**Table:**

C_8_H_20_N^+^·C_9_H_16_B_11_^−^	*Z* = 2
*M**_r_* = 373.38	*F*(000) = 400
Triclinic, *P*1	*D*_x_ = 1.031 Mg m^−^^3^
Hall symbol: -P 1	Melting point: 433 K
*a* = 8.8201 (6) Å	Mo *K*α radiation, λ = 0.71073 Å
*b* = 12.0929 (11) Å	Cell parameters from 4623 reflections
*c* = 12.1858 (11) Å	θ = 6.8–20.7°
α = 81.032 (7)°	µ = 0.05 mm^−^^1^
β = 79.899 (7)°	*T* = 293 K
γ = 71.553 (7)°	Block, colourless
*V* = 1206.82 (18) Å^3^	0.3 × 0.25 × 0.2 mm

### Data collection

**Table:**

Stoe Stadi CCD diffractometer	3228 reflections with *I* > 2σ(*I*)
Radiation source: fine-focus sealed tube	*R*_int_ = 0.051
graphite	θ_max_ = 25.0°, θ_min_ = 5.1°
ω scans	*h* = −10→10
17081 measured reflections	*k* = −14→14
4219 independent reflections	*l* = −14→14

### Refinement

**Table:**

Refinement on *F*^2^	Secondary atom site location: difference Fourier map
Least-squares matrix: full	Hydrogen site location: difference Fourier map
*R*[*F*^2^ > 2σ(*F*^2^)] = 0.048	H atoms treated by a mixture of independent and constrained refinement
*wR*(*F*^2^) = 0.101	*w* = 1/[σ^2^(*F*_o_^2^) + (0.01*P*)^2^ + 0.45*P*] where *P* = (*F*_o_^2^ + 2*F*_c_^2^)/3
*S* = 1.00	(Δ/σ)_max_ < 0.001
4219 reflections	Δρ_max_ = 0.16 e Å^−^^3^
363 parameters	Δρ_min_ = −0.15 e Å^−^^3^
0 restraints	Extinction correction: *SHELXL97* (Sheldrick, 2008), Fc^*^=kFc[1+0.001xFc^2^λ^3^/sin(2θ)]^-1/4^
Primary atom site location: structure-invariant direct methods	Extinction coefficient: 0.037 (3)

### Special details

**Table:**

Geometry. All e.s.d.'s (except the e.s.d. in the dihedral angle between two l.s. planes) are estimated using the full covariance matrix. The cell e.s.d.'s are taken into account individually in the estimation of e.s.d.'s in distances, angles and torsion angles; correlations between e.s.d.'s in cell parameters are only used when they are defined by crystal symmetry. An approximate (isotropic) treatment of cell e.s.d.'s is used for estimating e.s.d.'s involving l.s. planes.
Refinement. Refinement of *F*^2^ against ALL reflections. The weighted *R*-factor *wR* and goodness of fit *S* are based on *F*^2^, conventional *R*-factors *R* are based on *F*, with *F* set to zero for negative *F*^2^. The threshold expression of *F*^2^ > σ(*F*^2^) is used only for calculating *R*-factors(gt) *etc*. and is not relevant to the choice of reflections for refinement. *R*-factors based on *F*^2^ are statistically about twice as large as those based on *F*, and *R*- factors based on ALL data will be even larger.

### Fractional atomic coordinates and isotropic or equivalent isotropic displacement parameters (Å^2^)

**Table:**

	*x*	*y*	*z*	*U*_iso_*/*U*_eq_	
N1	0.02767 (14)	0.15270 (10)	0.23820 (10)	0.0443 (3)	
C11	0.1693 (2)	0.11482 (16)	0.30484 (17)	0.0572 (4)	
H11A	0.145 (2)	0.1795 (17)	0.3523 (15)	0.075 (6)*	
H11B	0.264 (2)	0.1153 (15)	0.2471 (15)	0.067 (5)*	
C12	0.1927 (2)	−0.00116 (17)	0.37487 (17)	0.0756 (6)	
H12A	0.0981	0.0009	0.4282	0.115 (5)*	
H12B	0.2842	−0.0170	0.4139	0.115 (5)*	
H12C	0.2110	−0.0617	0.3274	0.115 (5)*	
C13	0.0403 (3)	0.06144 (18)	0.16256 (17)	0.0621 (5)	
H13A	0.034 (2)	−0.0082 (16)	0.2173 (15)	0.071 (5)*	
H13B	−0.055 (2)	0.0949 (16)	0.1246 (15)	0.074 (6)*	
C14	0.1930 (3)	0.0312 (2)	0.08048 (18)	0.0937 (7)	
H14A	0.1895	−0.0262	0.0355	0.149 (6)*	
H14B	0.2844	0.0001	0.1208	0.149 (6)*	
H14C	0.2024	0.1005	0.0329	0.149 (6)*	
C15	0.0310 (3)	0.26924 (16)	0.17111 (18)	0.0655 (5)	
H15A	0.021 (2)	0.3226 (17)	0.2329 (15)	0.074 (6)*	
H15B	0.140 (2)	0.2534 (16)	0.1284 (16)	0.075 (6)*	
C16	−0.0984 (3)	0.32222 (19)	0.09677 (17)	0.0891 (7)	
H16A	−0.0897	0.2691	0.0434	0.126 (5)*	
H16B	−0.0861	0.3948	0.0577	0.126 (5)*	
H16C	−0.2023	0.3366	0.1416	0.126 (5)*	
C17	−0.12997 (19)	0.16523 (15)	0.31589 (15)	0.0494 (4)	
H17A	−0.119 (2)	0.0852 (16)	0.3536 (14)	0.062 (5)*	
H17B	−0.207 (2)	0.1839 (14)	0.2658 (14)	0.060 (5)*	
C18	−0.1717 (2)	0.25361 (16)	0.39823 (15)	0.0660 (5)	
H18A	−0.1861	0.3305	0.3586	0.101 (4)*	
H18B	−0.0860	0.2361	0.4430	0.101 (4)*	
H18C	−0.2697	0.2509	0.4459	0.101 (4)*	
C1	0.8578 (2)	−0.42097 (13)	0.37589 (14)	0.0537 (4)	
H1	0.905 (2)	−0.5044 (15)	0.4091 (13)	0.064 (5)*	
B2	0.7262 (3)	−0.39328 (16)	0.28148 (17)	0.0561 (5)	
H2	0.6959 (19)	−0.4675 (14)	0.2554 (13)	0.064 (5)*	
B3	0.9243 (2)	−0.38352 (16)	0.23998 (17)	0.0555 (5)	
H3	1.012 (2)	−0.4499 (15)	0.1888 (13)	0.065 (5)*	
B4	0.9825 (2)	−0.33524 (16)	0.35199 (17)	0.0535 (5)	
H4	1.108 (2)	−0.3739 (14)	0.3728 (13)	0.064 (5)*	
B5	0.8195 (2)	−0.31531 (15)	0.46186 (16)	0.0516 (5)	
H5	0.8448 (19)	−0.3415 (14)	0.5490 (14)	0.062 (5)*	
B6	0.6607 (2)	−0.35139 (16)	0.41916 (17)	0.0560 (5)	
H6	0.588 (2)	−0.3994 (14)	0.4814 (13)	0.065 (5)*	
B7	0.7589 (2)	−0.27118 (15)	0.19136 (16)	0.0503 (4)	
H7	0.7377 (18)	−0.2580 (13)	0.1044 (13)	0.057 (4)*	
B8	0.5950 (2)	−0.25187 (16)	0.30200 (17)	0.0523 (5)	
H8	0.465 (2)	−0.2256 (14)	0.2866 (13)	0.067 (5)*	
B9	0.6529 (2)	−0.20266 (15)	0.41506 (16)	0.0496 (4)	
H9	0.5641 (19)	−0.1430 (14)	0.4736 (13)	0.057 (4)*	
B10	0.8521 (2)	−0.19299 (14)	0.37266 (15)	0.0463 (4)	
H10	0.8952 (18)	−0.1288 (13)	0.4032 (12)	0.054 (4)*	
B11	0.9180 (2)	−0.23517 (15)	0.23469 (16)	0.0486 (4)	
H11	1.002 (2)	−0.1994 (14)	0.1743 (13)	0.063 (5)*	
B12	0.7131 (2)	−0.15303 (14)	0.27349 (14)	0.0436 (4)	
C2	0.64183 (18)	−0.02308 (13)	0.22826 (12)	0.0467 (4)	
C3	0.58462 (17)	0.07904 (13)	0.19784 (12)	0.0446 (4)	
C4	0.51356 (16)	0.20251 (12)	0.16824 (12)	0.0420 (3)	
C5	0.46196 (19)	0.27790 (14)	0.25139 (15)	0.0503 (4)	
H5A	0.4757 (19)	0.2488 (14)	0.3277 (14)	0.059 (5)*	
C6	0.3917 (2)	0.39677 (15)	0.22449 (18)	0.0633 (5)	
H6A	0.357 (2)	0.4489 (17)	0.2854 (16)	0.078 (6)*	
C7	0.3720 (2)	0.44060 (16)	0.11525 (18)	0.0662 (5)	
H7A	0.318 (2)	0.5237 (19)	0.0958 (16)	0.089 (6)*	
C8	0.4210 (2)	0.36716 (16)	0.03291 (18)	0.0647 (5)	
H8A	0.408 (2)	0.3951 (16)	−0.0451 (16)	0.074 (5)*	
C9	0.4921 (2)	0.24830 (15)	0.05830 (15)	0.0536 (4)	
H9A	0.530 (2)	0.1947 (15)	−0.0011 (14)	0.062 (5)*	

### Atomic displacement parameters (Å^2^)

**Table:**

	*U*^11^	*U*^22^	*U*^33^	*U*^12^	*U*^13^	*U*^23^
N1	0.0430 (7)	0.0412 (7)	0.0525 (7)	−0.0178 (6)	−0.0068 (6)	−0.0043 (5)
C11	0.0394 (9)	0.0633 (11)	0.0709 (12)	−0.0172 (8)	−0.0107 (9)	−0.0057 (9)
C12	0.0600 (12)	0.0723 (13)	0.0766 (13)	0.0020 (10)	−0.0135 (10)	0.0039 (10)
C13	0.0695 (13)	0.0592 (11)	0.0616 (11)	−0.0210 (10)	−0.0051 (10)	−0.0189 (9)
C14	0.1051 (18)	0.0901 (16)	0.0746 (14)	−0.0207 (13)	0.0179 (13)	−0.0270 (12)
C15	0.0741 (13)	0.0518 (10)	0.0705 (12)	−0.0272 (10)	−0.0061 (11)	0.0080 (9)
C16	0.1112 (18)	0.0705 (13)	0.0693 (13)	−0.0084 (12)	−0.0202 (13)	0.0107 (10)
C17	0.0387 (9)	0.0518 (10)	0.0590 (10)	−0.0155 (7)	−0.0070 (8)	−0.0048 (8)
C18	0.0640 (12)	0.0628 (11)	0.0675 (11)	−0.0110 (9)	−0.0052 (9)	−0.0162 (9)
C1	0.0603 (10)	0.0321 (8)	0.0662 (10)	−0.0067 (7)	−0.0200 (8)	−0.0001 (7)
B2	0.0654 (13)	0.0385 (9)	0.0701 (12)	−0.0170 (9)	−0.0233 (10)	−0.0037 (9)
B3	0.0587 (12)	0.0423 (10)	0.0620 (12)	−0.0061 (9)	−0.0080 (10)	−0.0137 (9)
B4	0.0475 (11)	0.0417 (9)	0.0684 (12)	−0.0036 (8)	−0.0184 (9)	−0.0056 (8)
B5	0.0625 (12)	0.0394 (9)	0.0506 (10)	−0.0089 (8)	−0.0179 (9)	0.0007 (8)
B6	0.0575 (12)	0.0431 (10)	0.0645 (12)	−0.0158 (9)	−0.0070 (10)	0.0033 (9)
B7	0.0608 (12)	0.0420 (9)	0.0505 (10)	−0.0138 (8)	−0.0177 (9)	−0.0041 (8)
B8	0.0461 (11)	0.0437 (10)	0.0675 (12)	−0.0125 (8)	−0.0165 (9)	0.0008 (8)
B9	0.0509 (11)	0.0395 (9)	0.0523 (10)	−0.0063 (8)	−0.0056 (9)	−0.0030 (8)
B10	0.0515 (10)	0.0353 (8)	0.0540 (10)	−0.0106 (8)	−0.0171 (8)	−0.0035 (7)
B11	0.0454 (10)	0.0439 (9)	0.0554 (11)	−0.0124 (8)	−0.0055 (8)	−0.0056 (8)
B12	0.0453 (10)	0.0358 (8)	0.0496 (10)	−0.0092 (7)	−0.0126 (8)	−0.0035 (7)
C2	0.0472 (9)	0.0422 (9)	0.0512 (9)	−0.0130 (7)	−0.0127 (7)	−0.0004 (7)
C3	0.0393 (8)	0.0413 (8)	0.0530 (9)	−0.0125 (7)	−0.0115 (7)	0.0025 (7)
C4	0.0329 (7)	0.0374 (7)	0.0552 (9)	−0.0127 (6)	−0.0097 (6)	0.0057 (7)
C5	0.0447 (9)	0.0457 (9)	0.0557 (10)	−0.0120 (7)	−0.0045 (7)	0.0034 (7)
C6	0.0558 (11)	0.0453 (9)	0.0826 (13)	−0.0113 (8)	−0.0001 (10)	−0.0065 (9)
C7	0.0557 (11)	0.0400 (9)	0.0959 (15)	−0.0129 (8)	−0.0150 (10)	0.0160 (10)
C8	0.0676 (12)	0.0563 (11)	0.0693 (12)	−0.0228 (9)	−0.0242 (10)	0.0227 (10)
C9	0.0554 (10)	0.0491 (9)	0.0581 (10)	−0.0189 (8)	−0.0156 (8)	0.0055 (8)

### Geometric parameters (Å, °)

**Table:**

N1—C13	1.511 (2)	B3—H3	1.106 (17)
N1—C17	1.5173 (19)	B4—B5	1.767 (3)
N1—C11	1.519 (2)	B4—B10	1.768 (2)
N1—C15	1.521 (2)	B4—B11	1.772 (3)
C11—C12	1.498 (2)	B4—H4	1.112 (17)
C11—H11A	0.991 (19)	B5—B10	1.764 (2)
C11—H11B	0.996 (19)	B5—B9	1.768 (3)
C12—H12A	0.9600	B5—B6	1.771 (3)
C12—H12B	0.9600	B5—H5	1.101 (16)
C12—H12C	0.9600	B6—B8	1.771 (3)
C13—C14	1.509 (3)	B6—B9	1.771 (3)
C13—H13A	1.000 (19)	B6—H6	1.117 (17)
C13—H13B	0.973 (19)	B7—B12	1.773 (2)
C14—H14A	0.9600	B7—B11	1.777 (3)
C14—H14B	0.9600	B7—B8	1.778 (3)
C14—H14C	0.9600	B7—H7	1.087 (15)
C15—C16	1.501 (3)	B8—B12	1.779 (2)
C15—H15A	1.041 (19)	B8—B9	1.791 (3)
C15—H15B	0.98 (2)	B8—H8	1.127 (17)
C16—H16A	0.9600	B9—B10	1.778 (3)
C16—H16B	0.9600	B9—B12	1.782 (3)
C16—H16C	0.9600	B9—H9	1.118 (16)
C17—C18	1.497 (2)	B10—B12	1.777 (2)
C17—H17A	0.987 (17)	B10—B11	1.778 (3)
C17—H17B	0.942 (17)	B10—H10	1.101 (15)
C18—H18A	0.9600	B11—B12	1.785 (3)
C18—H18B	0.9600	B11—H11	1.092 (16)
C18—H18C	0.9600	B12—C2	1.548 (2)
C1—B5	1.692 (2)	C2—C3	1.202 (2)
C1—B4	1.698 (3)	C3—C4	1.4393 (19)
C1—B2	1.698 (2)	C4—C5	1.389 (2)
C1—B3	1.699 (3)	C4—C9	1.389 (2)
C1—B6	1.703 (3)	C5—C6	1.385 (2)
C1—H1	1.010 (17)	C5—H5A	0.956 (16)
B2—B8	1.762 (3)	C6—C7	1.373 (3)
B2—B7	1.767 (3)	C6—H6A	0.993 (19)
B2—B3	1.767 (3)	C7—C8	1.366 (3)
B2—B6	1.773 (3)	C7—H7A	0.98 (2)
B2—H2	1.118 (16)	C8—C9	1.383 (2)
B3—B7	1.767 (3)	C8—H8A	0.973 (18)
B3—B11	1.769 (3)	C9—H9A	0.990 (17)
B3—B4	1.775 (3)		
			
C13—N1—C17	106.34 (12)	C3—C2—B12	177.15 (16)
C13—N1—C11	110.83 (14)	C2—C3—C4	176.64 (17)
C17—N1—C11	110.72 (13)	C5—C4—C9	118.84 (14)
C13—N1—C15	111.48 (14)	C5—C4—C3	119.59 (14)
C17—N1—C15	111.00 (13)	C9—C4—C3	121.55 (14)
C11—N1—C15	106.54 (13)	C6—C5—C4	120.32 (16)
C12—C11—N1	115.71 (14)	C7—C6—C5	120.04 (19)
C14—C13—N1	115.43 (16)	C8—C7—C6	120.15 (17)
C16—C15—N1	115.55 (16)	C7—C8—C9	120.53 (18)
C18—C17—N1	116.43 (13)	C8—C9—C4	120.11 (18)
			
C2—C3—C4—C5	8(3)	C5—C6—C7—C8	0.2 (3)
C2—C3—C4—C9	−171 (3)	C6—C7—C8—C9	−0.5 (3)
C9—C4—C5—C6	−0.6 (2)	C7—C8—C9—C4	0.2 (3)
C3—C4—C5—C6	−179.29 (14)	C5—C4—C9—C8	0.3 (2)
C4—C5—C6—C7	0.4 (3)	C3—C4—C9—C8	178.98 (15)
